# Cerebellar Predominant Increase in mRNA Expression Levels of Sirt1 and Sirt3 Isoforms in a Transgenic Mouse Model of Huntington’s Disease

**DOI:** 10.1007/s11064-020-03069-0

**Published:** 2020-06-10

**Authors:** Andras Salamon, Rita Maszlag-Török, Gábor Veres, Fanni Annamária Boros, Evelin Vágvölgyi-Sümegi, Anett Somogyi, László Vécsei, Péter Klivényi, Dénes Zádori

**Affiliations:** 1 Department of Neurology, Interdisciplinary Excellence Center, Faculty of Medicine, Albert Szent-Györgyi Clinical Center, University of Szeged, Semmelweis u. 6, Szeged, 6725 Hungary; 2 MTA-SZTE Neuroscience Research Group of the Hungarian Academy of Sciences, Budapest, Hungary

**Keywords:** Huntington’s disease, Transgenic, Sirt1, Sirt3, Sirtuin, Brain

## Abstract

The potential role of Sirt1 and Sirt2 subtypes of Sirtuins (class III NAD^+^-dependent deacetylases) in the pathogenesis of Huntington’s disease (HD) has been extensively studied yielding some controversial results. However, data regarding the involvement of Sirt3 and their variants in HD are considerably limited. The aim of this study was to assess the expression pattern of Sirt1 and three Sirt3 mRNA isoforms (Sirt3-M1/2/3) in the striatum, cortex and cerebellum in respect of the effect of gender, age and the presence of the transgene using the N171-82Q transgenic mouse model of HD. Striatal, cortical and cerebellar Sirt1-Fl and Sirt3-M1/2/3 mRNA levels were measured in 8, 12 and 16 weeks old N171-82Q transgenic mice and in their wild-type littermates. Regarding the striatum and cortex, the presence of the transgene resulted in a significant increase in Sirt3-M3 and Sirt1 mRNA levels, respectively, whereas in case of the cerebellum the transgene resulted in increased expression of all the assessed subtypes and isoforms. Aging exerted minor influence on Sirt mRNA expression levels, both in transgene carriers and in their wild-type littermates, and there was no interaction between the presence of the transgene and aging. Furthermore, there was no difference between genders. The unequivocal cerebellar Sirtuin activation with presumed compensatory role suggests that the cerebellum might be another key player in HD in addition to the most severely affected striatum. The mitochondrially acting Sirt3 may serve as an interesting novel therapeutic target in this deleterious condition.

## Introduction

Huntington’s disease (HD) is an autosomal dominant neurodegenerative disease [66]. HD is caused by expansion of CAG repeats in the *IT15* gene encoding Huntington protein (Htt) which has an important role in the maintenance of cellular energy metabolism and mitochondrial function [50]. Previous works demonstrated that mutant Huntington protein (mHtt) inhibits the function of a key metabolic master regulator, namely peroxisome proliferator-activated receptor-gamma coactivator 1α (PGC-1α), which, amongst others, has an essential role in mitochondrial biogenesis [8, 25].

Sirtuins are class III NAD^+^-dependent deacetylases [38]. Currently there are seven identified mammalian Sirtuin subtypes (SIRT1-7), which are localized in different cellular compartments (nuclear: SIRT1 (the mammalian orthologue of the yeast Silent information regulator 2 protein (Sir2)), -6, -7; mitochondrial: SIRT3, -4, -5; cytoplasmatic: SIRT2) [36]. In addition to the above-detailed subtypes, alternative splicing results in further isoforms of Sirtuins [31, 67]. Several molecular targets of Sirtuins, including the above-mentioned PGC-1α, were identified as participants of the regulation of energy metabolism, circadian rhythm, stress response, apoptosis and aging [38]. The association between SIRTs and neurodegenerative disorders, including HD, has been widely studied using these models [2, 22, 24, 27, 36, 56]. Calorie restriction is capable of increasing SIRT1 protein level in the brain, liver, hearth and white adipose tissue of mice [39], and also increases the lifespan in the N171-82Q transgenic (tg) mouse model of HD [11]. In contrast to these findings, exercise, which induces the expression of Sirt3-M1 and -M2 isoforms [47], did not elongate the lifespan in the same mouse model of HD [43]. Regarding Sirt1 mRNA and SIRT1 protein expression changes in HD the results are somewhat inconsistent: SIRT1 protein levels were found to be reduced in human brain tissue and in the R6/1 transgenic mouse model of HD as well [18, 41]. Tulino et al. found that SIRT1 activity becomes reduced in R6/2 (with a mean CAG repeat number (MRN) of 204) and *Hdh*Q150 (MRN: 165) mice, in the background of which they hypothesized altered phosphorylation status of SIRT1 via an 5′ AMP-activated protein kinase α1-related mechanism in the striatum and cerebellum [61]. Although the presence of the transgene did not affect either Sirt1 mRNA, or SIRT1 protein expression in the striatum of R6/2 mice, there was a significant decrease in mRNA expression from 4 to 9 weeks in wild-type (wt) animals [61]. In contrast to these findings, cerebellar Sirt1 mRNA expression increased significantly by 9 and 14 weeks of age in R6/2 mice, whereas SIRT1 protein levels significantly decreased predominantly in wt mice by 14 weeks of age. In *Hdh*Q150 and wt mice the SIRT1 protein level did not show any alteration either in the 2 or in the 22 months old animals [61]. A later whole-brain study reported the elevation of Sirt1 mRNA level in 5, 8 and 11 weeks old R6/2 mice (MRN: 144). However, up-regulation of Sirt1 was only observed in their 8, but not in 12 weeks old counterparts possessing a mean CAG repeat size of 182 [45]. Regarding SIRT1 protein expression, there was a consistent elevation in tg mice with 182 mean CAG repeats in both ages, and when examined at 12 weeks of age, without gender differences [45]. Besides the somewhat inconsistent findings obtained from the above-mentioned studies, the experiments influencing the expression of SIRT1 or of its orthologues from a therapeutic point of view yielded more controversial results. Parker et al. were the first who demonstrated that *Sir2* overexpression and resveratrol (RESV) treatment (one of the most important non-selective Sirtuin inducer) could delay the development of neuronal dysfunction in a *Caenorhabditis elegans* model of HD (Htt N-terminal fragment, 128Q) in vivo [42]. They also reported that RESV prevented the striatal neuronal cell death in *Hdh*Q111 knock-in mice [42]. To test the potential neuroprotective effect of SIRT1, Jeong et al. crossed a brain-specific *Sirt1* knockout mice (BSKO; genotype: *Sirt1*^flox/flox^) with the R6/2 HD model mice [22]. They detected the exacerbation of the neuropathological aspects of HD indicated by lower striatal (neuronal) volume [22], whereas they found the opposite in SIRT1 overexpressing knock-in mice (Sirt1-KI-R6/2). These animals showed longer survival time (30% extension) and less prominent neuropathological alterations [22]. They proposed that the neuroprotective effect of SIRT1 is exerted through the activation of the cyclic AMP response element binding transcription factor-regulated transcription coactivator 1 factor, which leads to the enhancement of the brain-derived neurotropic factor-mediated neuroprotection [22]. Jiang et al. crossed *Sirt1* and N171-82Q or BAC HD transgenic mice which resulted in offsprings with decelerated disease progression and reduction of brain atrophy probably via the overexpression of *Sirt1* [24]. In contrast to these findings, the pharmacological inhibition of SIRT1 by selisistat exerted beneficial effects in both Drosophila and mouse models of HD and was found to be safe in human studies as well [52, 54].

SIRT2, another member of the Sirtuin family, is suspected to enhance the disease process in HD. Chopra et al. reported a beneficial effect of SIRT2 inhibition in R6/2 HD mice [7]. Previously published articles demonstrated that there is an age-dependent SIRT2 accumulation which results in microtubule deacetylation in mouse brain and spinal cord [33]. These alterations lead to the disruption of microtubule-associated cellular transport which is an important component of the pathogenesis of HD [10, 16]. However, it seems that the ablation of SIRT2 did not prevent the development of HD-related pathological mechanisms in R6/2 mice [5].

Similar to SIRT1, for which most of the results support a protective role in HD, SIRT3 is also proposed to have a beneficial effect regarding the pathogenesis of the disease [38], though the available data are limited. SIRT3 is involved in the regulation of fatty acid oxidation, urea- and amino acid pathways [2]. Striatal administration of a RESV dimer (ε-viniferin treatment) reduced ROS level through SIRT3-mediated superoxide dismutase 2 (SOD2) induction in striatal progenitor cells (*Hdh*(Q111)) [14]. Some authors suggested that SIRT3 could alleviate the pathological process in HD through the deacetylation of the mitochondrial complexes (I, II, V) as well [1, 2]. Furthermore, SIRT3 is implicated in autophagy regulation via its effect on chaperones [28].

Regarding the role of another Sirtuin isoforms (SIRT4-7) in the pathogenesis of HD, experimental data are lacking.

The aim of the current study was to further elucidate the role Sirt1 and Sirt3 isoforms with presumed beneficial effects in HD, because the available data are somewhat controversial regarding Sirt1, and no deep assessment was done regarding the M1, M2 and M3 isoforms of Sirt3. For that purpose the authors applied a multi-dimensional approach to simultaneously assess the effect of the transgene, the time course of the disease and their interaction as well in addition to the screening of regional and possible gender-related differences in the N171-82Q tg mouse model of HD.

## Materials and Methods

### Animals

8, 12 and 16 weeks old N171-82Q and as their control, B6C3 wt mice with identical genetic background (female and male animals distributed equally) were involved in this study (n = 6–7 in each group). The HD model mice originally came from Jackson Laboratories (USA). They were housed in cages under standard conditions with 12–12 h light-dark cycle. The food and water were freely available. The experiments were carried out in accordance with European Communities Council Directive (86/609/EEC) and were approved by the local animal care committee. All animals were euthanized via isoflurane overdose (Forane; Abott Laboratories Hungary Ltd., Budapest, Hungary).

### Sample Handling

The brains were rapidly removed from the skull and both hemispheres were dissected into the following brain areas: striatum, cortex and cerebellum. The tissue samples were stored at − 80 °C until the RT-PCR analysis.

### RT-PCR Analysis

To perform the RT-PCR analysis, total RNA was isolated from the striatal, cortical and cerebellar samples with Trizol reagent according to manufacturer’s protocol (Molecular Research Center, USA). The RNA concentration was determined with MaestroNano spectrophotometer. The RNA integrity was certified by 1% gel electrophoresis. For cDNA synthesis 1 µg of total RNA, random hexamer primers and reverse transcriptase were used (Revert Aid First Strand cDNA Synthesis Kit; Thermo Scientific, USA). The synthesized cDNA was stored at − 20 °C. Real-time PCR analysis (CFX 96 Real Time System; Bio-Rad, USA) with various Sirtuin primers was performed in 20 µl final volume using syber green label (PCR Biosystems, USA) [47]. Primer sequences and the exact thermal cycling conditions are described in our previous work [47]. We used the 18S rRNA as endogenous control (Applied Biosystems, USA). To calculate the relative mRNA expression levels, we used the 2^−∆∆Ct^ method [30].

### Statistics

**Fig. 1:**
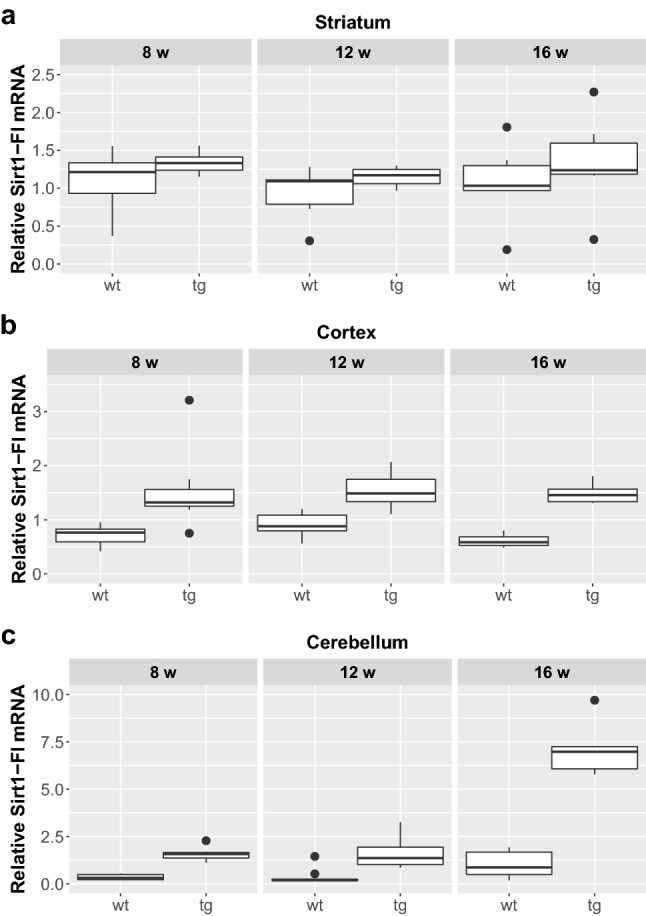
Relative mRNA expression level of Sirt1-Fl in the striatum (**a**), cortex (**b**) and cerebellum (**c**) of N171-82Q transgenic and B6C3 wild-type mice of three age groups. The Sirt1-Fl level significantly elevated in all cortical and cerebellar samples in each age group of tg animals compared to wt mice. Aging caused a significant increase only in the cerebellum of tg group by 16 weeks of age. For clarity, the levels of significance were indicated separately in Fig. 5 in a special table format. Values are plotted as medians and interquartile ranges; *tg* = transgenic, *wt* = wild-type, *w* = weeks

**Fig. 2:**
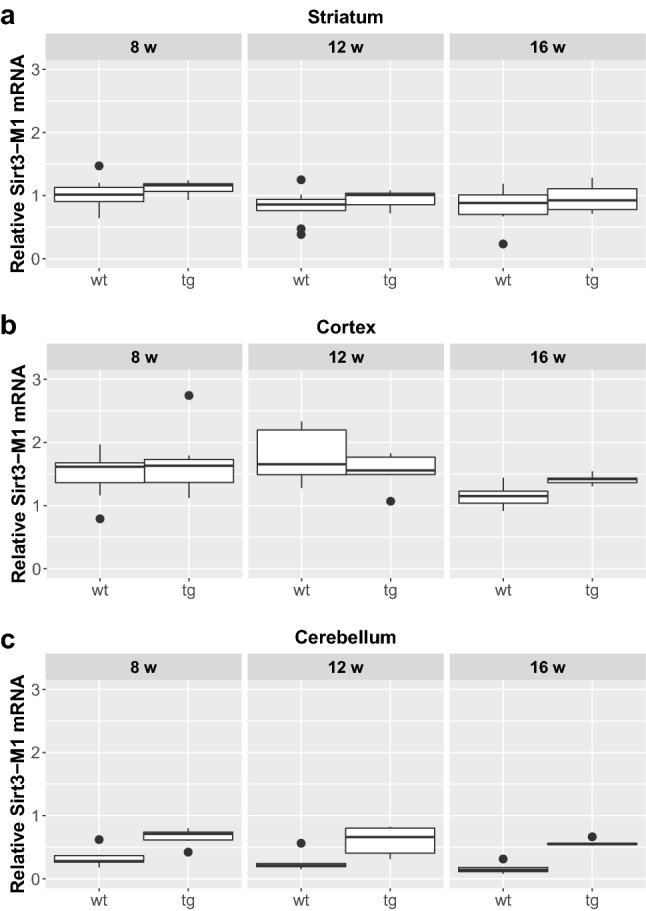
Relative mRNA expression level of Sirt3-M1 in the striatum (**a**), cortex (**b**) and cerebellum (**c**) of N171-82Q transgenic and B6C3 wild-type mice of three age groups. The Sirt3-M1 level was significantly elevated in all cerebellar samples in each age group of tg animals compared to wt mice. For clarity, the levels of significance were indicated separately in Fig. 5 in a special table format. Values are plotted as medians and interquartile ranges; *tg* = transgenic, *wt* = wild-type, *w* = weeks

**Fig. 3:**
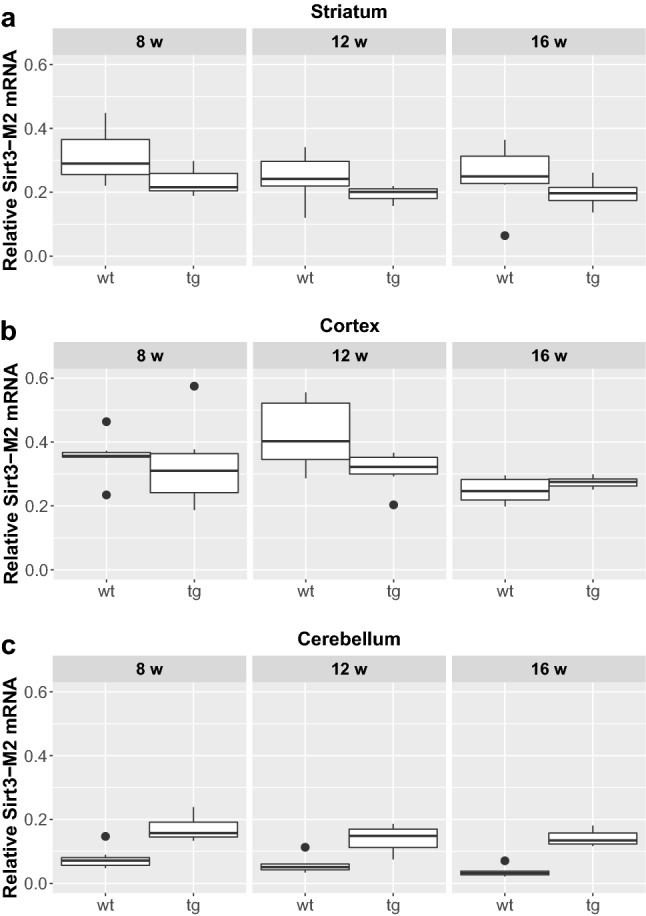
Relative mRNA expression level of Sirt3-M2 in the striatum (**a**), cortex (**b**) and cerebellum (**c**) of N171-82Q transgenic and B6C3 wild-type mice of three age groups. The Sirt3-M2 level was significantly elevated in all cerebellar samples in each age group of tg animals compared to wt mice. Aging caused a significant decrease only in the cortex of wt group by 16 weeks of age. For clarity, the levels of significance were indicated separately in Fig. 5 in a special table format. Values are plotted as medians and interquartile ranges; *tg* = transgenic, *wt* = wild-type, *w* = weeks

**Fig. 4:**
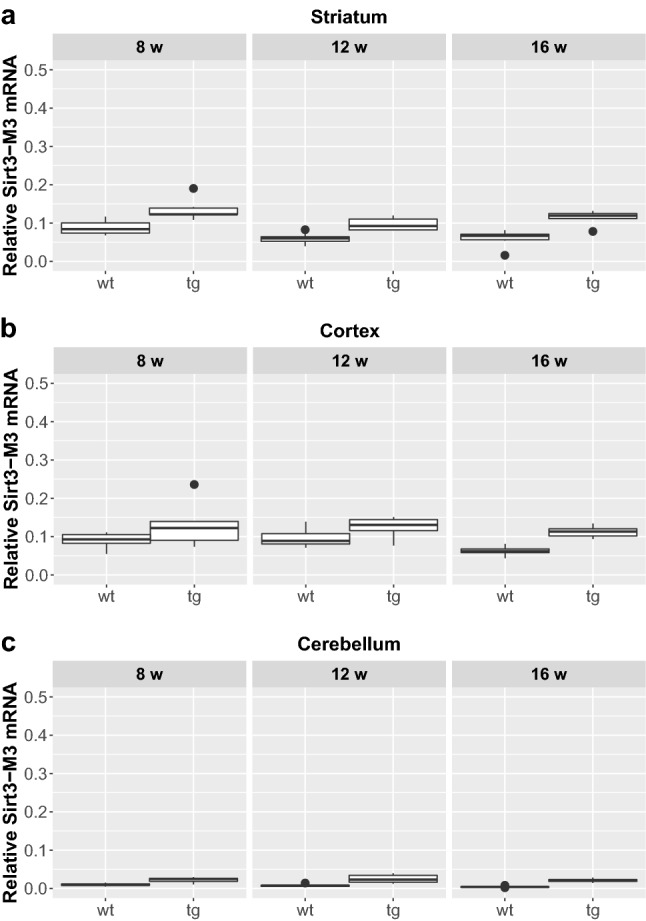
Relative mRNA expression level of Sirt3-M3 in the striatum (**a**), cortex (**b**) and cerebellum (**c**) of N171-82Q transgenic and B6C3 wild-type mice of three age groups. The Sirt3-M3 level was significantly elevated in all striatal and cerebellar samples in each age group of tg animals compared to wt mice. The striatal expression decreased significantly by 12 weeks of age in both wt and tg animals. For clarity, the levels of significance were indicated separately in Fig. 5 in a special table format. Values are plotted as medians and interquartile ranges; *tg* = transgenic, *wt* = wild-type, *w* = weeks

**Fig. 5:**
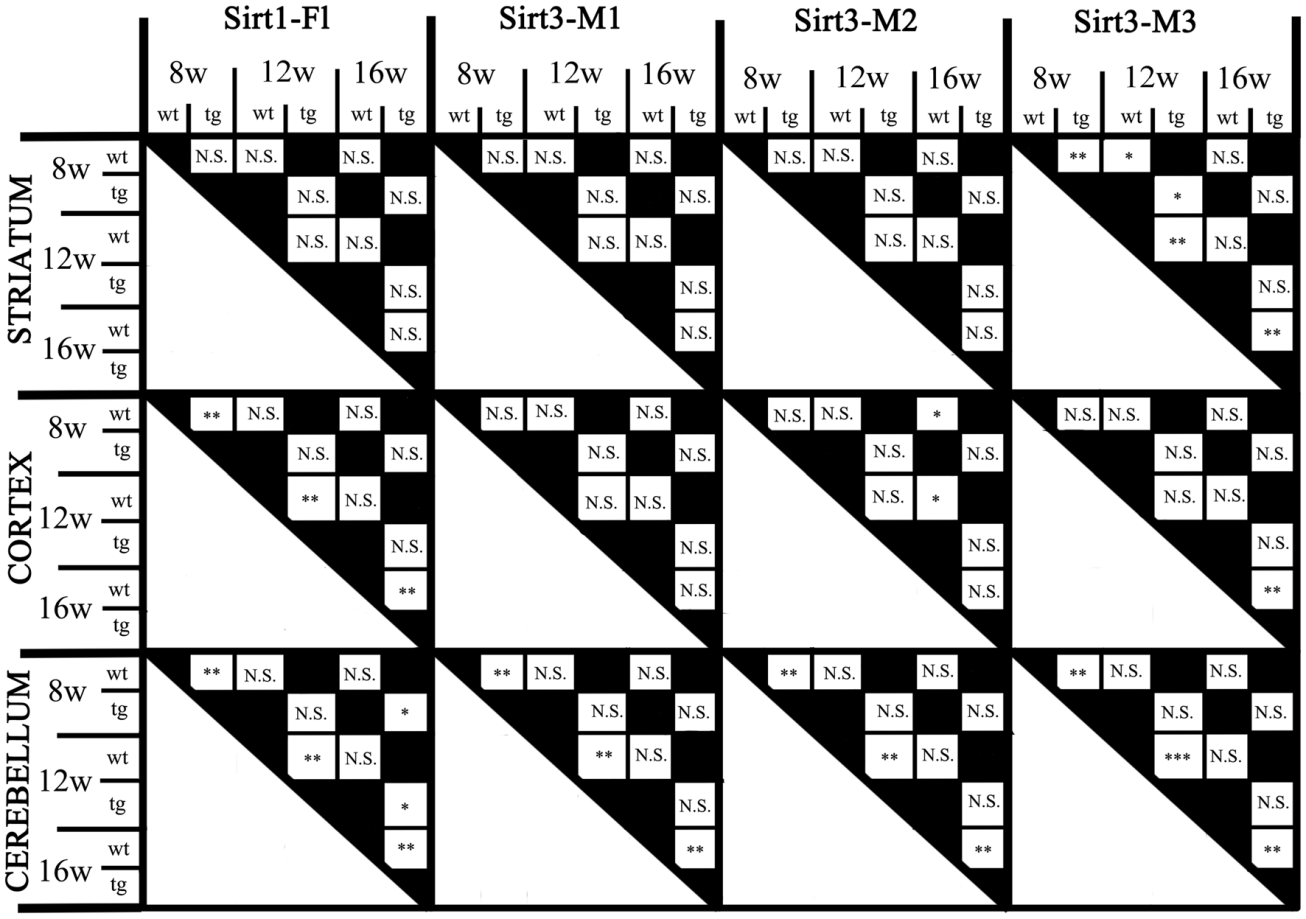
The statistical delineation of the effect of the transgene and the time course of the neurodegenerative process on the expression pattern of Sirt1 and Sirt3 isoforms. In addition to the presented statistical differences, there were no significant interactions between the presence of the transgene and age. *p < 0.05, **p < 0.01, ***< 0.001; *N.S*. = not significant; *tg* = transgenic, *wt* = wild-type, *w* = weeks

All statistical calculations were performed with the use of the freely available R software (R Development Core Team). First, the relative mRNA expression levels were calculated separately regarding each gene (i.e., Sirt1 or Sirt3), but they were normalized to the striatal level of the main subtype or isoform (i.e., Sirt1-Fl and Sirt3-M1) of 8 weeks old wt mice in each case to allow a time course analysis of the changes of expression patterns. Then we checked the distribution of data populations with the Shapiro–Wilk test and the homogeneity of variances with the Levene’s test. In several cases the data diverged from Gaussian distribution and the variances were not equal. For that reason, we applied the Sheirer-Ray-Hare test to determine the differences between the investigated factors and their interaction as well. Afterwards, we carried out permutation t-tests as post hoc analysis for pairwise comparison and Type I errors from multiple comparisons were controlled with false discovery rate. As some of the possible comparisons would not have yielded meaningful information regarding the a priori decided presumptions, a maximum of 9 pairwise comparisons were implemented in case of each subtype or isoform analyzed by each brain region. The results were considered significant when the corrected p values were greater than 0.05. The data were presented as median and 1st and 3rd quartiles (Figs. 1, 2, 3, 4, 5).

## Results

We could not detect any significant difference between male and female mice regarding the expression of any of the assessed Sirtuin isoforms either in the wt or in the tg groups, so they were pooled for further analyses. Furthermore, in respect of Sirt1 and Sirt3 expression, no interaction was found between the presence of the transgene and aging. Focusing on their separate effects, there was a significant elevation of Sirt1-Fl (full length) expression in all the cortical and cerebellar samples of 8, 12 and 16 weeks old tg animals compared to wt mice (cortex (8 weeks: p = 0.0029; 12 weeks: p = 0.0018; 16 weeks: p = 0.0029); cerebellum (8 weeks: p = 0.0052; 12 weeks: p = 0.0054; 16 weeks: p = 0.0065), but not in the striatum (Figs. 1 and 5). Regarding the effect of aging on Sirt1-Fl expression levels, we detected significant increase only in the cerebellum of tg group at 16 weeks of age (8 vs. 16 weeks: p = 0.0245; 12 vs. 16 weeks: p = 0.0316). There was no detectable change in Sirt3-M1 expression in the striatum and cortex of any age groups (8, 12, 16 weeks). In contrast, there was a clear elevation of Sirt3-M1 mRNA expression in cerebellar samples of all age groups of tg animals compared to wt mice (8 weeks: p = 0.0024; 12 weeks: = 0.0024; 16 weeks: p = 0.0024; Figs. 2 and 5). We could not observe any age-related effect in the Sirt3-M1 isoform in either group. Regarding Sirt3-M2 we could not detect any difference between wt and tg groups in the striatal and cortical samples, but we could identify a significant elevation in the cerebellum of tg animals in each age group compared to wt controls (8 weeks: p = 0.0021; 12 weeks: p = 0.0012; 16 weeks: p = 0.0021; Figs. 3 and 5). When assessing the effect of aging on Sirt3-M2 expression levels, we detected significant decrease only in the cortex of wt group at 16 weeks of age (8 vs. 16 weeks: p = 0.038; 12 vs. 16 weeks: p = 0.038). The expression of Sirt3-M3 elevated in each striatal and cerebellar tg groups, but only in the 16 weeks old group in the tg cortex compared to wt mice (striatum (8 weeks: p = 0.0097; 12 weeks: p = 0.0054; 16 weeks: p = 0.0097); cortex (16 weeks: p = 0.0032); cerebellum (8 weeks: p = 0.002; 12 weeks: p = 0.0006; 16 weeks: p = 0.0016) (Figs. 4 and 5). In the striatal Sirt3-M3 samples there was detectable decrease of expression by 12 weeks of age in both wt and tg animals (8 vs. 12 weeks (wt-wt): p = 0.0243; 8 vs. 12 weeks (tg–tg): p = 0.036).

## Discussion

HD is a neurodegenerative disorder, which has currently no curative treatment, but encouraging clinical trials are ongoing (e.g. antisense oligonucleotide treatment) [13, 56]. These therapeutic approaches target the pathological trinucleotide repeat expansion in the mutant hunting in mRNA, the extent of which has been demonstrated to have the strongest association with the age of disease onset and severity [12, 26, 37]. However, other important influencing factors of the disease have already been identified as well, which may serve as good candidates for the augmentation of beneficial effects obtained by the currently available therapies [32, 64].

Sirtuins are surely involved in the neurodegenerative process in HD, however, there are controversial results regarding their role [36, 38, 45, 61]. Further studies are needed to clarify these controversies and to better explore the role of different Sirtuin subtypes and isoforms obtained by alternative splicing [2, 22, 24, 27, 36, 56]. Accordingly, we aimed at contributing to the clarification of the controversies regarding Sirt1 and Sirt3 mRNA expression patterns by a better characterization of region- and aging-specific changes in the mRNA expression levels of Sirt1 and three Sirt3 isoforms using the N171-82Q tg mouse model of HD.

Tulino et al. found a significant decrease in striatal Sirt1 mRNA expression from 4 to 9 weeks in the wt group, whereas cerebellar Sirt1 mRNA expression increased significantly by 9 and 14 weeks of age in the same control group in experiments with R6/2 mouse model of HD (MRN: 204) [61]. The presence of the transgene seemingly did not affect Sirt1 mRNA expression. Another research group (Reynolds et al.) measured Sirt1 mRNA levels in the whole-brain samples of 5, 8, 11- (MRN: 144) and 8, 12 weeks old (MRN: 182) R6/2 mice [45]. In the 5, 8 and 11 weeks old mice (MRN: 144) there was a significant increase in mRNA levels of all tg groups. Aging did not affect the values and accordingly there was no significant interaction between age and the presence of the transgene. Regarding the only female cohort with 182 mean CAG repeat size, the significant increase could be observed only in the 8 weeks old group [45]. Due to the different CAG repeats, ages, brain regions and gender composition the comparability of these results is limited. Therefore, we find it important to get closer to the unbiased preclinical modelling of changes in Sirt1 mRNA expression in HD. Thus, we paid a special attention to study design regarding the following points: we used the N171-82Q tg model of HD, because the phenotype of these mice mimics better the majority of human cases with considerable similarities in pathological alterations as well than the R6/2 tg mice, the symptoms of which mainly resemble that of juvenile HD cases. Furthermore, the authors have extensive previous experiences with this model [15, 35, 40, 59, 62, 63, 68, 69], which enables the drawing of indirect correlations between the characteristic features of disease progression with the time course of the changes in mRNA expression patterns. Characteristically, in N171-82Q mice the symptoms begin to develop at approximately 2 months of age and disease progression results in a mean survival time of 110–130 days [68, 69]. Accordingly, the study of mice at the age of 8, 12 and 16 weeks may represent early, moderate and advanced stages of the modelled disease. In addition to the assessment of the presence of transgene on Sirt mRNA expression changes in key structures (striatum, overlying cortex, cerebellum) of the regulation of motor functions, our study design involved the determination of the effect of aging and its interaction with the presence of the transgene with a view on gender-related effects as well. First of all, we found no significant differences between genders regarding either of the above-mentioned aspects, and therefore gender issues seemingly did not introduce bias into the studies of Tulino et al. and Reynolds et al. [45, 61]. Similarly to the study of Tulino et al. [61], we found no effect of the transgene in the striatum regarding either age groups, but a marked increase in Sirt1 expression was demonstrated in cortical and cerebellar samples of tg animals compared to wt controls in all age groups similar to that found by Reynolds et al. [45] applying whole brain samples. The magnitude of difference increased only by 16 weeks of age, and again, similarly to the latter study, no significant interaction was found between aging and the presence of the transgene. Aging-related increase in Sirt1 mRNA expression either in the striatal or cerebellar samples of wt mice, found by Tulino et al. [61], could not be confirmed by the current study.

Although data suggest that the induction of mitochondrially acting SIRT3 may be capable of exerting beneficial effects in a HD model [14], the expression pattern of Sirt3 mRNA isoforms has never been studied in any HD model. Similar to that found in case of Sirt1-Fl, a remarkable increase of cerebellar expression of all Sirt3 isoforms could be observed in tg animals compared to wt controls in the current study. The striatal expression of Sirt-M3 in all age groups and the cortical expression of Sirt3-M3 by 16 weeks of age were found to be increased. However, we have to note here that the relative expression level of Sirt3-M3 mRNA is considerably lower compared to that of the other two isoforms. The expression level of cortical Sirt3-M2 in wt mice and striatal Sirt3-M3 in wt and tg mice decreased by 16 and 12 weeks of age, respectively.

The pattern of expression changes in the cerebellum regarding any of the assessed Sirt subtypes and isoforms strongly resembles to that of PGC-1α expression changes (either of its full length or N-terminal fragment) as we have shown in a previous study using the same animal model of HD [58]. The reason behind the same pattern may be that Sirtuins are upstream regulators of PGC-1α expression [8, 25]. Similar cerebellar predominant PGC-1α activation was induced by long-term physical exercise [47] and by 1-methyl-4-phenyl-1,2,3,6-tetrahydropyridine (MPTP) treatment in its acute phase [60], but not by cooling, by short-term exercise [47], or by 3-nitropropionic acid (3-NP), the toxin used for modeling HD-associated striatal damage [58, 65].

Although the cerebellum is known not to be the primarily affected structure in HD, there is increasing evidence of its involvement in the pathomechanism of the disorder [48]. A considerable loss of Purkinje cells was demonstrated in some HD patients with predominant motor symptoms [51], the extent of which may become more pronounced in patients with higher CAG repeat number [19]. However, there is no clear relationship between the disease stage and the degree of Purkinje and granular cell loss, and the degree of cerebellar degeneration is quite variable [17, 23, 46]. The exact background of this variability, involving the sparing of alterations as well in some human cases, is not known and needs further elucidation. Nevertheless, some studies proved cerebellar hypermetabolism in HD [44] with a presumed compensatory role for dysfunction in the fronto-striato-thalamic motor circuit [9, 53]. The significant elevations in cerebellar PGC-1α [58] and Sirt mRNA expressions in the N171-82Q HD model may be considered as an important part of this compensatory cerebellar hypermetabolism. Furthermore, the increased Sirt3 mRNA expression indicates the involvement of mitochondrial activation as well. The beneficial role of the Sirt – PGC-1α axis in intact cerebellar functioning may be further supported by the finding that FL-PGC-1α knockout mice demonstrated reactive astrogliosis in cerebellar nuclei, whereas the striatum and cortex were almost totally spared [55]. Although data obtained from the above-mentioned toxin studies are somewhat controversial, this may be resolved by considering that in case of 3-NP the most vulnerable structures are the striatum and the hippocampus, and not the cerebellum [34], whereas MPTP is capable of inducing cerebellar degeneration as well in addition to its well-known deleterious effects on the nigrostriatal system [57]. However, the mRNA expression changes in Sirt1 and Sirt3 levels following MPTP intoxication were not significant either in its acute or chronic phase (unpublished data).

Regarding genotype-phenotype correlations, the first description of the applied N171-82Q transgenic mouse model of HD has already demonstrated that in addition to cortical, hippocampal, amygdalar and striatal involvement, an expressed deposition of intranuclear inclusions were present in cerebellar granule cells as well [49]. It was also verified by the authors, but no remarkable neuronal loss or reactive astrogliosis was found [40]. This may be explained by that the presence of abundant intranuclear inclusions does not compulsorily equals to severe neuronal damage, indeed, it was presumed to exert protective effects [3]. This may further support our hypothesis that coping mechanisms in the cerebellum, including inclusion body formation and enhanced expression pattern of the Sirt – PGC-1α axis, demonstrated by the current and previous studies [59] of the authors, may be capable of exerting rather neuroprotective than damaging effects. Accordingly, the progression of the decrease of coordination, which was described as one of the key features of this model and probably attributed to cerebellar dysfunction, could be observed from 3 months of age on [20]. However, the hypokinetic feature of N171-82Q transgenic mice, rather explained by the dysfunction of the basal ganglia, involving the striatum, was still present at 8 weeks of age [69]. The evidence from unbiased anatomical work-ups of the authors also favors the above-detailed hypothesis, i.e., more than 20% of striatal neurons contained intranuclear inclusions at 16 weeks of age, whereas no remarkable neuronal loss, but prominent neuronal atrophy could be detected at this age, responsible for the decrease of striatal volume and brain weight [69]. However, besides the well demonstrated atrophy of striatum, cortex, hippocampus, hypothalamus, thalamus and amygdala, the volume of the cerebellum was still preserved even at 18 weeks of age [6].

The characteristic pattern observed in preclinical findings could not be confirmed on human samples, where Sirt1, -2, and -3 mRNA levels were assessed, demonstrating elevated cortical and striatal Sirt1 and striatal Sirt2 expression in HD patients without any change regarding Sirt3 or the cerebellum [4]. However, this gene expression study was carried out on post-mortem samples obtained from multiple centers, and the postmortem delay of sample handling and other characteristics of the specimens were not given, so their effect on individual differences in mRNA decay cannot be ruled out introducing a bias into the results. Nevertheless, further human studies are needed to be able to better determine the human relevance of the current preclinical findings.

The major limitation of the current study is the lack of experiments on protein expression changes of the corresponding Sirtuins. However, the widely applied antibody-based methods in this topic (e.g. Western blotting) are semiquantitative at best, mostly lacking appropriate sensitivity and specificity [29]. Although the application of deep proteomic analysis by mass spectrometry is appropriate, but quite challenging [21], especially in case of brain samples with considerably high lipid content. Accordingly, this was out of the scope of the current study. Nevertheless, future experiments are needed to confirm the results of the present work at proteome level.

In conclusion, the current study demonstrates an unequivocal elevation of Sirt1 mRNA expression level in the cortical and cerebellar samples of HD transgenic animals compared to their age-matched wild-type littermates. An increase of these differences with aging could be observed only in the cerebellum of transgenic animals, presumably related to disease progression. Regarding the expression pattern of the mitochondrially acting Sirt3 isoforms (M1, M2, M3), which have not been assessed before in HD, a similar transgene-specific increase of their cerebellar level was observed, and only the striatum showed a likewise elevation of exclusively the M3 isoform regarding the other 2 assessed brain regions. Accordingly, the observed pattern of changes with a presumed compensatory role may draw attention to that the involvement of cerebellum in HD may be more pronounced than previously thought, yielding a novel target for therapeutic approaches aiming at symptom relief in that deleterious condition.
